# Continuous gastric saline perfusion elicits cardiovascular responses in freshwater rainbow trout (*Oncorhynchus mykiss*)

**DOI:** 10.1007/s00360-021-01408-3

**Published:** 2021-10-07

**Authors:** Daniel Morgenroth, Tristan McArley, Andreas Ekström, Albin Gräns, Michael Axelsson, Erik Sandblom

**Affiliations:** 1 Department of Biological and Environmental Sciences, University of Gothenburg, PO Box 463, 405 30 Gothenburg, Sweden; 2 Department of Animal Environment and Health, Swedish University of Agricultural Sciences, 532 23 Skara, Sweden

**Keywords:** Gastrointestinal sensing mechanisms, Vascular resistance, Blood flow, Salinity

## Abstract

When in seawater, rainbow trout (*Oncorhynchus mykiss*) drink to avoid dehydration and display stroke volume (SV) mediated elevations in cardiac output (CO) and an increased proportion of CO is diverted to the gastrointestinal tract as compared to when in freshwater. These cardiovascular alterations are associated with distinct reductions in systemic and gastrointestinal vascular resistance (*R*_Sys_ and *R*_GI_, respectively). Although increased gastrointestinal blood flow (GBF) is likely essential for osmoregulation in seawater, the sensory functions and mechanisms driving the vascular resistance changes and other associated cardiovascular changes in euryhaline fishes remain poorly understood. Here, we examined whether internal gastrointestinal mechanisms responsive to osmotic changes mediate the cardiovascular changes typically observed in seawater, by comparing the cardiovascular responses of freshwater-acclimated rainbow trout receiving continuous (for 4 days) gastric perfusion with half-strength seawater (½ SW, ~ 17 ppt) to control fish (i.e., no perfusion). We show that perfusion with ½ SW causes significantly larger increases in CO, SV and GBF, as well as reductions in *R*_Sys_ and *R*_GI_, compared with the control, whilst there were no significant differences in blood composition between treatments. Taken together, our data suggest that increased gastrointestinal luminal osmolality is sensed directly in the gut, and at least partly, mediates cardiovascular responses previously observed in SW acclimated rainbow trout. Even though a potential role of mechano-receptor stimulation from gastrointestinal volume loading in eliciting these cardiovascular responses cannot be excluded, our study indicates the presence of internal gastrointestinal milieu-sensing mechanisms that affect cardiovascular responses when environmental salinity changes.

**Supplementary Information:**

The online version contains supplementary material available at 10.1007/s00360-021-01408-3.

## Introduction

Successful transitions from freshwater (FW) to seawater (SW) by euryhaline fishes rely on a series of behavioural and physiological adjustments to switch from hyper- to hypo-osmoregulation, respectively (Zydlewski and Wilkie 2013). These adjustments compensate for the passive fluxes of water and ions across permeable biological membranes, the direction of which reverse upon transition between salinities (Kültz 2015; Marshall and Grosell 2005). In FW, fish compensate for the loss of salts and osmotic gain of water by increasing branchial and dietary salt absorption, as well as by elevating glomerular filtration rate and dilute urine production (Larsen et al. 2014). Conversely, in marine environments, teleost fishes face a constant diffusive loss of water and ion uptake (Larsen et al. 2014). To prevent dehydration, marine fishes drink large amounts of water, which is then absorbed in the intestine coupled to NaCl uptake (Grosell 2006; Grosell and Taylor 2007). Excess absorbed monovalent ions are excreted branchially, while excretion of divalent ions is either renally or rectally mediated (Edwards and Marshall 2013; Evans et al. 2005).

Following a transition from FW to SW, several cardiovascular changes are also known to occur. Rainbow trout (*Oncorhynchus mykiss*) acutely transferred to SW display stroke volume (SV) mediated elevations in cardiac output (CO; Maxime et al. 1991), and in trout chronically acclimated to SW, these elevations in SV are associated with an increased central venous pressure and thus cardiac filling pressure (Brijs et al. 2016b). Concomitantly, SW-acclimated rainbow trout have a reduced dorsal aortic blood pressure (*P*_DA_) compared with FW conspecifics (Sundell et al. 2018; Morgenroth et al. 2019; Olson and Hoagland 2008). Blood flow through a vascular bed or individual vessel (e.g., ventral aorta) can be expressed as the quotient of the pressure difference across the vasculature (e.g., difference between *P*_DA_ and central venous pressure) divided by the vascular resistance (e.g., systemic vascular resistance, *R*_Sys_; Olson 1998; Sandblom and Gräns 2017). Indeed, SW-acclimated trout have a reduced *R*_Sys_ that explains the increased CO along with reduced *P*_DA_ (Sundell et al. 2018). In addition, Olson and Hoagland (2008) showed that SW-acclimated rainbow trout were unable to fully compensate for the dehydrating effect of the hyperosmotic milieu and were in a state of chronic hypovolemia, which could also contribute to reduce arterial blood pressure in SW.

To facilitate the convection of absorbed water and ions, and possibly to improve O_2_ supply to the gastrointestinal tissues, fish transitioning from FW to SW elevate blood flow in the celiacomesenteric artery that perfuses the gastrointestinal tract. For example, the gastrointestinal blood flow (GBF) of rainbow trout doubled 96 h following acute SW-transfer (Brijs et al. 2015). Furthermore, rainbow trout chronically acclimated to SW had elevated CO and distributed an increased proportion of CO to the gastrointestinal tract (GBF/CO) to sustain an increased GBF (Brijs et al. 2016a). These elevations in GBF are driven by substantial reductions in gastrointestinal vascular resistance (*R*_GI_), which likely contribute to the lower *R*_Sys_ of trout in SW (Sundell et al. 2018; Morgenroth et al. 2019). However, although an increased GBF is likely crucial to maintain osmoregulation in hyperosmotic environments, very little is known regarding the mechanisms and internal stimuli contributing to the reduced *R*_GI_ in euryhaline fishes adjusting to marine conditions.

In mammals, internal stimuli eliciting elevations in GBF have been extensively studied, particularly in light of post-prandial hyperaemia (for reviews see Gallavan and Chou 1985; Matheson et al. 2000). A stimulus known to induce gastrointestinal vasodilation in mammals is intestinal hyperosmolality (Bohlen 1982, 1998; Levine et al. 1978; VanHeerden et al. 1968). Given that teleosts in SW drink substantial quantities of hyperosmotic fluid, elevated gastrointestinal osmolality can be hypothesised to induce gastrointestinal hyperemia and other distinctive cardiovascular changes that occur in teleosts during SW transition and acclimation. SW ingested by teleost fish is typically quickly desalinated in the oesophagus (Parmelee and Renfro 1983; Nagashima and Ando 1994; Takei et al. 2017; Brijs et al. 2015), such that the concentration of Na^+^ and Cl^−^ in the fluid entering the stomach is approximately halved (Grosell 2006). Thus, the osmolality of the fluid reaching the stomach is still hyperosmotic relative to the fish (~ 500 vs*.* ~ 300 mOsm L^−1^, respectively), after which it is progressively processed along the anterior intestine reaching a salinity that is approximately equivalent to the cellular and plasma osmolality (Shehadeh and Gordon 1969; Taylor and Grosell 2006; Kirsch and Meister 1982; Parmelee and Renfro 1983; Tsukada et al. 2005). One possibility, therefore, is that gastrointestinal hyperaemia in fish in SW is mediated directly via a mechanism sensitive to changes in osmolality within the stomach or anterior intestine.

Given the importance of drinking to maintain body fluid balance in SW, and following the premise that the amount of gastrointestinal blood supply must be coupled to drinking rate to facilitate the absorption and convection of water and ions, we tested the hypothesis that the cardiovascular responses during SW acclimation result from increased intestinal osmolality. Specifically, to circumvent the potential confounding effects of variations in environmental salinity and examine the direct internal stimuli from the imbibed water, we simulated SW drinking by infusing half-strength SW (½ SW) directly into the stomach lumen of rainbow trout kept in FW while continuously recording cardiovascular variables.

## Methods

### Experimental animals

Rainbow trout (*Oncorhynchus mykiss*, see Table 1 for body mass and morphometric details) were obtained from a local hatchery (Vänneåns Fiskodling AB, Halland, Sweden) and kept in FW at 10–11 °C for a minimum of four weeks prior to the start of the experiments. Fish were housed in 2000 L tanks with aerated recirculating FW and a 12:12 h light:dark photoperiod, and fed dry commercial pellets (7 mm, Protec Trout pellets, Skretting, Norway) twice a week. Experimental fish were fasted for 3 days prior to surgery to ensure they were in a post-absorptive state. All experimental procedures were covered by ethical permits 165–2015 and 5.8.18–10907/2020, approved by the regional ethical committee in Gothenburg.

**Table 1:** Morphometric variables of rainbow trout (*Oncorhynchus mykiss*) experimental treatment groups

Measured variables	Control	½ SW
Body mass (g)	764.9 ± 38.1 (8)	789.9 ± 105.7 (8)
Fork length (mm)	400.9 ± 4.7 (8)	399.0 ± 12.3 (8)
Condition factor	1.19 ± 0.05 (8)	1.20 ± 0.04 (8)
Relative spleen mass (%)	0.35 ± 0.04 (7)	0.41 ± 0.05 (5)
Relative ventricular mass (%)	0.08 ± 0.00 (8)	0.08 ± 0.00 (7)
Percentage compact myocardium (%)	41.2 ± 3.1 (8)	41.1 ± 2.8 (7)

½ SW, half-strength seawater. All values are means ± SEM. Sample sizes are indicated within parentheses for each treatment group. There were no significant differences (*P* < 0.05) between treatment groups, as assessed by independent samples *t*-tests

### Surgery and instrumentation

**Fig. 1:**
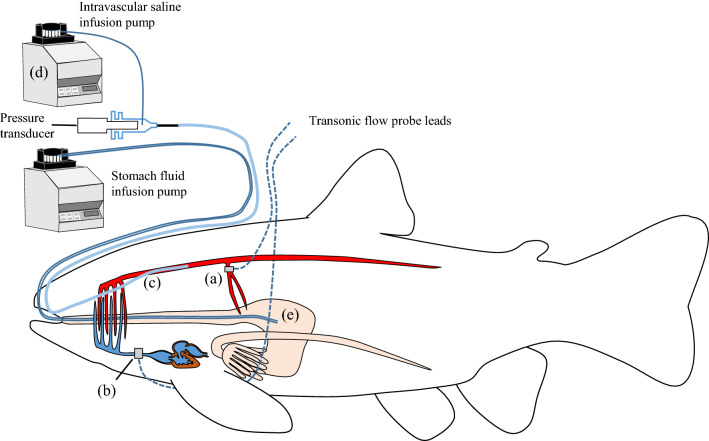
Schematic drawing of experimental setup and fish instrumentation. Rainbow trout (*Oncorhynchus mykiss*) was instrumented with a 1.5 PSB Transonic blood flow probe around the celiacomesenteric artery for measurements of gastrointestinal blood flow (**a**), a ventral aortic 2.5 PSL blood flow probe for measurements of cardiac output (**b**) and a dorsal aortic catheter for blood pressure measurements (**c**). The catheter was attached to a pressure transducer that had a PE-10 catheter inserted via a side arm allowing for the continuous slow infusion (7.5 µl h^−1^) of heparinized saline (0.9% NaCl) to optimize catheter patency using a peristaltic pump (**d**). The trout were also instrumented with a gastric catheter connected to a peristaltic pump for continuous infusion (5.4 ± 0.2 ml kg^−1^ h^−1^) of half-strength seawater (½ SW, ~ 17 ppt, **e**). Control fish were instrumented identically, although remained non-perfused

Fish were anesthetized in FW containing 150 mg L^−1^ MS-222 (Tricaine methanesulfonate, Scan Aqua AS, Årnes, Norway) buffered with 300 mg L^−1^ NaHCO_3_. Following measurements of fork length and body mass, the fish were placed on their left side on a surgery table covered with wet foam. Anaesthesia was maintained throughout the surgery by irrigating the gills with aerated FW at 10 °C containing 75 mg L^−1^ MS-222 and 150 mg L^−1^ NaHCO_3_. First, the celiacomesenteric artery, which originates from the dorsal aorta (Fig. 1; Olson 2011; Sandblom and Gräns 2017), was accessed via a lateral incision performed in the abdominal wall ~ 3 mm above the pectoral fin. The vessel was then dissected free with care to not damage any surrounding vessels or nerves. A Transonic flow probe (1.5 PRB; Transonic Systems, Ithaca, NY) was placed around the vessel, allowing for measurements of GBF. The incision was closed with interrupted 3-0 silk sutures and the lead was secured with a 2-0 silk suture in front of the dorsal fin. The ventral aorta was then accessed through a small incision in the isthmus and fitted with a perivascular Transonic flow probe (2.5 PSL; Transonic Systems, Ithaca, NY) for recordings of CO. The probe was secured with two sutures around the probe lead; one close to the probe head inside the opercular cavity and another in the skin immediately outside the opercular cavity. The probe lead was placed below the pectoral fin and secured with the same 2-0 suture as the celiacomesenteric flow probe lead.

Next, to allow for P_DA_ recordings and blood sampling, the dorsal aorta was cannulated with a PE-50 catheter following the descriptions of Soivio et al. (1975). Briefly, a hole was made through the snout and a PE-160 catheter, heat flared at one end, was inserted. A custom-made PE-50 catheter with a bubble ~10 cm from the tip was inserted into the dorsal aorta using a sharp steel wire as guide. To verify correct placement of the catheter, blood was withdrawn and adequate blood pressure was confirmed. The end of the catheter opposite to the one inserted in the dorsal aorta was exteriorized through the PE-160, locking it in place against the bubble, following which the catheter was further secured with the dorsal 2-0 suture. The catheter was then flushed with heparinized (100 IU ml^−1^) 0.9% saline and sealed with a pin. In addition, the insertion wound was sealed with ethyl 2-cyanoacrylate. Finally, another PE-50 catheter was inserted into the stomach through the mouth to allow for continuous fluid perfusion as detailed below. The distance between the inserted end of the gastric catheter and the stop bubble was 8 cm, which ensured that the catheter opening was placed in the stomach lumen. This catheter was also exteriorized through the snout in a similar way as described above and tied to the dorsal suture.

After surgery, the fish were transferred to individual 12.5 L PVC tubes kept in a ~ 120 L experimental tank filled with aerated recirculating FW at 10–11 °C. To improve the patency of the blood pressure signal throughout the long experimental protocol (see below), the dorsal aortic catheter was connected to a pressure transducer with a PE-10 catheter side-arm. The PE-10 catheter was connected to a peristaltic pump (Gilson 312 Minipuls 3, Villiers-Le-Bel, France) that infused the fish with a constant flow of 7.5 µl h^−1^ of heparinized saline (0.9% NaCl, 25 IU ml^−1^ heparin) to prevent blood clot formation at the tip of the blood pressure catheter, thus improving the long-term quality of the blood pressure signal. Preliminary tests indicated that this low saline flow did not affect the blood pressure relative to fish without intra-vascular saline perfusion (data not shown). Fish were allowed to recover from surgery for > 48 h before the experimental protocol started. The surgical procedure and instrumentation was identical for all the fish used in the study. A summary of the instrumentations of the fish and the experimental setup is shown in Fig. 1.

### Experimental protocol

Following the > 48 h of recovery from surgery, routine cardiovascular variables were recorded for 24 h. Next, each individual was allocated into one of the two treatment groups where they were either continuously perfused with ½ SW for 96 h or left non-perfused (Control) for the same period of time. To mimic the intra-gastric water composition following oesophageal desalination in fish spontaneously drinking SW, fish in the seawater perfusion treatment (i.e. ½ SW) were perfused with full-strength SW (32–33 ppt) diluted 50:50 with deioinised water (i.e., ~ 17 ppt, 501 mOsm kg^−1^, 27.5 mS cm^−1^, [K^+^] 4.6 mmol L^−1^, [Na^+^] 209 mmol L^−1^, [Cl^−^] 227 mmol L^−1^, [Ca^2+^] 5.9 mmol L^−1^). Published estimated drinking rates in salmonids transferred to SW varies greatly throughout the acclimation period and typically ranges between 2.0 and 6.9 ml kg^−1^ h^−1^ (see Table 2). Generally, drinking rates gradually increase up until the first 3–4 days following acute transfer to SW (Usher et al. 1988; Fuentes and Eddy 1997b), although one study on small juvenile Atlantic salmon (*Salmo salar*, average body mass: 13.3 g) reported drinking rates of up to 25 ml kg^−1^ h^−1^ 8 h after SW transfer, before stabilizing at 5 ml kg^−1^ h^−1^ (Bath and Eddy 1979). It is unknown how drinking rate scales with size, and to the best of our knowledge, there are no studies of drinking rates for salmonids in the specific size range studied here. Nevertheless, to simulate drinking, the stomach was continuously perfused using a peristaltic pump (Gilson 312 Minipuls 3, Villiers-Le-Bel, France), which was individually adjusted to match an estimated mass-specific drinking rate of 5.4 ± 0.2 ml kg^−1^ h^−1^ as closely as possible, as this is the drinking rate previously determined for rainbow trout closest to the size of fish used in the current study (see Fig. 1 and Table 2, Shehadeh and Gordon 1969).

**Table 2:** Published values for drinking rates in Atlantic salmon (*Salmo salar*) and rainbow trout (*Oncorhynchus mykiss*) acclimated to seawater

Species	Size (g)	Salinity	Temperature (°C)	Drinking rate (ml kg^−1^ h^−1^)	Acclimation time (days)	References
*Salmo salar*	15–25	30–32 ppt	7–13	6.13	4	(Usher et al. 1988)
				6.88	9	
				6.22	86	
*Salmo salar*	50–70	891 mOsm L^−1^	6–13	4.0	7	(Fuentes and Eddy 1997a)
*Salmo salar*	50–70	894 mosm L^−1^	12–13	3.0	4	(Fuentes and Eddy 1997b)
				4.0	7	
*Salmo salar*	15–30	33 ppt	8–10	2.0	7	(Fuentes et al. 1996)
*Oncorhynchus mykiss*	20–30	28 ppt	8–10	2.0	7	
*Oncorhynchus mykiss*	150–250	32	17	5.4	10	(Shehadeh and Gordon 1969)
*Oncorhynchus mykiss*	50–150	31–32 ppt	12	2.1	> 30	(Bucking et al. 2011)

At the end of the protocol, a 1 ml blood sample was taken from the dorsal aortic catheter for analyses of haematocrit, haemoglobin concentration ([Haemoglobin]) and blood pH. The remaining blood in the sample was centrifuged for 5 min at 5000 g and the plasma was collected and frozen at − 80 °C for later analyses of osmolality and Na^+^, K^+^, Cl^+^ and Ca^2+^ concentrations. Fish was then euthanized and the wet masses of the spleen and heart ventricle (following the removal of the atrium, bulbus and blotting and emptying of blood) were determined. The ventricle was preserved in 70% ethanol for further analyses (see below). Finally, the stomach was opened to verify the correct position of the perfusion catheter.

### Data acquisition and calculations

Average cardiovascular variables were measured as one-hour means throughout the day, resulting in 24 daily measurements, although the length of the intervals were sometimes reduced in cases where the quality of the signal was reduced by spontaneous brief spurs of activity. Resting cardiovascular variables were subsequently determined as those occurring during periods with the lowest 20% of CO values each day.

The Transonic flow probes were connected to a Transonic blood flow meter (model T206; Transonic Systems, Ithaca, NY) and the signals where recorded using a PowerLab system (ADInstruments, Castle Hill, Australia) at a sampling rate of 10 Hz using LabChart Pro data acquisition software (version 7.3.2, AD Instruments, Castle Hill, Australia). To correct for any temperature effects on the Transonic flow probe readings, all probes were individually bench calibrated at the experimental temperature (10.5 °C), as specified by the user manual. The heart rate (HR) was calculated from the pulsatile blood flow traces using the cyclic measurements module in LabChart Pro. SV was calculated as:

$$\mathrm{SV}=\frac{\mathrm{CO}}{\mathrm{HR}}$$

*R*_Sys_ was calculated as:

$${R}_{\mathrm{Sys}}=\frac{{P}_{\mathrm{DA}}-\mathrm{central venous pressure}}{\mathrm{CO}}$$

assuming that central venous pressure is 0.

*R*_GI_ was calculated as:

$${R}_{\mathrm{GI}}=\frac{{P}_{\mathrm{DA}}-\mathrm{portal vein pressure}}{\mathrm{GBF}}$$

assuming portal vein pressure is 0.

The percentage of CO directed into the gastrointestinal tract was calculated as GBF/CO × 100. To further evaluate changes in cardiovascular variables within treatment groups the percent change from baseline (% Δ from baseline) was calculated as the proportional increase/decrease from baseline (defined as the values at day 0/prior to perfusion) and the last day of treatment (day 4).

Haematocrit was determined by spinning the blood in capillary tubes in a micro-haematocrit centrifuge for 5 min at 10,000 g and measuring the resulting fraction of red blood cells. [Haemoglobin] was measured using a handheld Hb 201 + analyser (Hemocue, Ängelholm, Sweden), and the values were corrected for fish blood according to Clark et al. (2008). The mean corpuscular haemoglobin concentration (MCHC) was calculated as:

$$\mathrm{MCHC}=\frac{[\mathrm{Haemoglobin}]}{\mathrm{Haematocrit}}\times 100$$

Plasma Na^+^, K^+^, Cl^−^ and Ca^2+^ concentrations were determined with an electrolyte analyzer (Convergys® ISE Comfort, Convergent Technologies, Coelbe, Germany). Plasma osmolality was determined using an Advanced Model 3320 micro-osmometer (Advanced Instruments, Norwood, MA, USA).

Condition factor for individual fish was calculated as:

$$\mathrm{Condition factor}=\frac{\mathrm{Body mass}}{{\mathrm{Fork length}}^{3}}\times 100$$

The relative ventricle mass was calculated as wet mass of the ventricle/body mass × 100. The proportion of ventricular compact myocardium was estimated by manually separating the compact and spongy muscle layers, drying overnight and weighing in accordance with methodological descriptions of Farrell et al. (2007). The percentage compact myocardium was then calculated as the dry mass of compact myocardium/dry mass of ventricle × 100. The relative spleen mass was calculated as wet mass of the spleen/body mass × 100.

### Statistical analyses

Statistical analyses were performed using SPSS statistics 24 for Windows (IBM Corp., Armonk, NY, USA). For all cardiovascular variables, to evaluate dynamic treatment effects on cardiovascular function, absolute values across treatment from day 0 to 4 are reported, as well as % Δ from baseline to further evaluate proportional changes in cardiovascular variables within treatment groups. Significant differences between treatments in cardiovascular variables at day 0 (i.e. prior to perfusion) and the % Δ from baseline, as well as morphometric and blood variables, were analysed using independent samples *t* tests or a Mann–Whitney *U* test if data were not normally distributed. A two-way repeated measures ANOVA was performed to compare cardiovascular performance during perfusion (i.e. day 1–4). Treatment (control or ½ SW) was specified as the between-subjects factor and time as within-subjects factor. Body mass was included as a covariate in the statistical model, but subsequently removed if no significant effect was found. Homogeneity of variances, normal distribution, equality of covariance matrices and normal distribution of the residuals were assessed by Levene’s test, Shapiro–Wilk’s test, Box’s *M* test and visual inspection of *Q*–*Q* plots, respectively. If the assumption of sphericity was violated as indicated by Mauchly’s Test, the data were analysed using Greenhouse–Geisser adjusted *F*-Tests. GBF was square root transformed to comply with the assumption of equality of covariance matrices. Equality of covariance matrices for R_Sys_ could not be achieved via transformations, therefore, the analysis was performed without including day 1 for this variable. Similarly, equality of variances of HR during day 4 could not be achieved via transformations, therefore this day was not included in the analysis of this variable. GBF/CO was transformed to its natural logarithm to comply with the assumption of normal distribution and CO was transformed to its natural logarithm to comply with the assumption of equality of variances and covariance matrices. Statistical significance was accepted at *P* < 0.05. All data are presented as means ± SE.

## Results

### Morphometric and cardiovascular variables before gastrointestinal perfusion

Body characteristics (body mass, length and condition factor) did not differ between treatment groups (Table 1). Similarly, routine cardiovascular variables did not differ significantly between treatment groups prior to the start of the perfusion protocol.

### Effects of gastrointestinal perfusion on cardiovascular variables

**Fig. 2:**
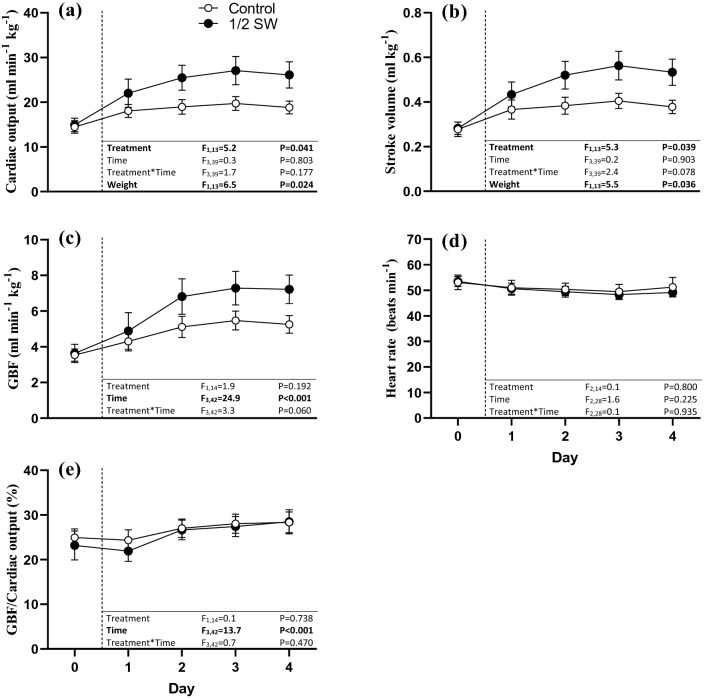
Circulatory responses of rainbow trout (*Oncorhynchus mykiss*) exposed to gastric seawater perfusion for 4 days. The figure illustrates **a** cardiac output (CO), **b** stroke volume (SV), **c** gastrointestinal blood flow (GBF) and **d** the proportion of cardiac output directed to the gut (GBF/CO). Fish were either non-perfused (Control, *n* = 8, open symbols) or gastrically perfused with half-strength seawater (½ SW, *n* = 7–8, ~ 17 ppt, filled symbols). The results from the two-way repeated measures ANOVA for the respective variables are presented in each panel. Differences between groups at day 0 were assessed using independent samples *t*-tests. Statistically significant differences are accepted at *P* < 0.05. All values are means ± SEM

Following the start of gastric perfusion, CO was higher in the ½ SW treatment group (mean CO during days 1–4: 18.9 ± 0.7 and 25.2 ± 1.5 ml kg^−1^ h^−1^ in control and ½ SW, respectively; Fig. 2a), and the increase between day 0 and day 4 was more than twofold greater in the ½ SW treatment relative to the control (% Δ from baseline; *t*(14) = − 2.381, *P* = 0.032; Fig. 4). The elevated CO observed in ½ SW was mediated by a significantly larger SV (Fig. 2b) as HR did not differ between treatments (Fig. 2d). Consistent with the greater increase in CO from baseline in the ½ SW treatment group, the % Δ from baseline in SV was also significantly greater in this group (*t*(14) = − 2.546, *P* = 0.023, Fig. 4).

**Fig. 3:**
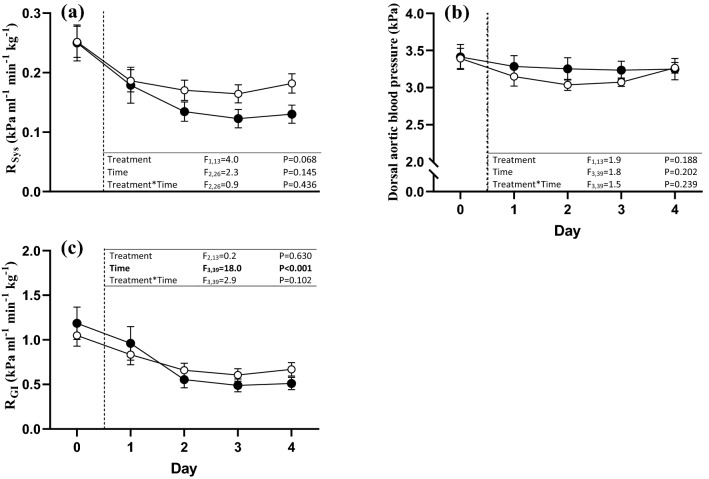
Vascular responses of rainbow trout (*Oncorhynchus mykiss*) exposed to gastric seawater perfusion for 4 days. The figure illustrates **a** systemic vascular resistance (*R*_Sys_), **b** dorsal aortic blood pressure (*P*_DA_) and **c** gastrointestinal vascular resistance (*R*_GI_). Fish were either non-perfused (Control, *n* = 7–8, open symbols) or gastrically perfused with half-strength seawater (½ SW, *n* = 6–8, ~ 17 ppt, filled symbols). The results from the two-way repeated measures ANOVA for the respective variables are presented in each panel. Differences between groups at day 0 were assessed using independent samples *t*-tests. Statistically significant differences are accepted at *P* < 0.05. All values are means ± SEM

**Fig. 4:**
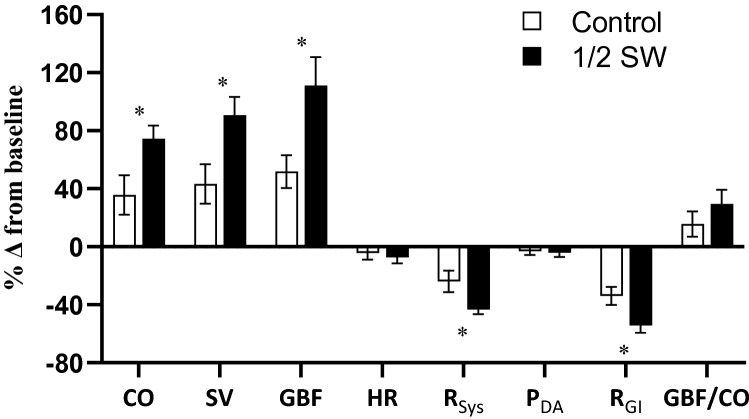
Percentage changes (%∆) from baseline (day 0) of the cardiovascular variables on day 4 of gastric perfusion in rainbow trout (*Oncorhynchus mykiss*). Fish were either non-perfused (Control, white bars) or gastrically perfused with half-strength seawater (½ SW, ~ 17 ppt, black bars). Abbreviations are: % Δ from baseline, percentage increase/decrease from baseline (day 0) at day 4 of perfusion; CO cardiac output, SV stroke volume, GBF gastrointestinal, HR heart rate, R_Sys_ systemic vascular resistance, P_DA_ dorsal aortic blood pressure, R_GI_ gastrointestinal vascular resistance, GBF/CO proportion of CO directed to the gastrointestinal tract. Statistically significant (*P* < 0.05) differences between treatments were determined via independent samples *t*-tests. *Indicates statistically significant differences between treatments. All values are means ± SEM. Sample sizes are: control, *n* = 8 and ½ SW, *n* = 6–8

The elevated CO in the ½ SW treatment was associated with a trend for a reduced *R*_Sys_ (Fig. 3a), while P_DA_ remained stable across experimental days and treatment groups (Fig. 3b). Again, the % Δ from baseline in *R*_Sys_ was significantly more pronounced in the ½ SW treatment compared to the control (treatment effect: *t*(9.451) = 2.384, *P* = 0.040; Fig. 4). GBF increased significantly throughout the protocol across experimental treatments, and was associated with a trend for an interaction between treatment and time (Fig. 2c), as the increase in GBF between day 0 and day 4 was more than double in the ½ SW than in the control treatment (% Δ from baseline; *t*(14) = − 2.632, *P* = 0.020; Fig. 4). The increases in GBF observed in both treatments were associated with general reductions in *R*_GI_ (Fig. 3c), and, although no treatment effect was identified, a trend for an interaction between treatment and time was found as reductions in *R*_GI_ were larger between day 0 and day 4 in the ½ SW treatment (% Δ from baseline, *t*(13) = 2.514, *P* = 0.026; Fig. 4). GBF/CO increased across treatments throughout the experimental protocol but there were no significant treatment effects (Figs. 2e and 4).

### Effects of gastrointestinal perfusion on cardiac morphology and blood parameters

Neither cardiac morphology (relative ventricular mass, *t*(13) = − 1.353, *P* = 0.199; and % compact myocardium, *t*(13) = 0.010, *P* = 0.992), nor relative spleen mass *t*(10) = − 1.009, *P* = 0.337) differed between treatment groups at the end of the experimental protocol (Table 1). Moreover, gastric perfusion had no significant effects on plasma osmolality (*t* (6.344) = − 1.359, *P* = 0.221), [K^+^] (*t*(11) = − 1.415, *P* = 0.185), [Na^+^] (*U* = 24, *P* = 0.731), [Ca^2+^] (*t*(11) = − 1.100, *P* = 0.295) or pH (*t*(10) = 1.248, *P* = 0.240), although there was a trend towards a higher [Cl^−^] in the ½ SW treatment (*t*(10) = − 2.190, *P* = 0.053; Table 3). Similarly, there were no significant differences in haematological status between experimental groups (haematocrit, (*U* = 29.5, *P* = 0.234); [haemoglobin], *t*(11) = − 0.534, *P* = 0.604; and MCHC, *t*(11) = − 1.124, *P* = 0.285; Table 3).

**Table 3:** Haematological and plasma variables in control and ½ SW treated rainbow trout (*Oncorhynchus mykiss*)

Measured variables	Control	½ SW
Haematocrit (%)	16.4 ± 0.9 (6)	18.5 ± 1.7 (7)
[Haemoglobin] (g L^−1^)	49.2 ± 3.6 (6)	52.8 ± 5.4 (7)
Mean corpuscular [haemoglobin] (g L^−1^)	298.8 ± 7.8 (6)	284.7 ± 9.4 (7)
pH	7.77 ± 0.04 (6)	7.71 ± 0.04 (6)
Osmolality (mOsm Kg^−1^)	295.3 ± 1.1 (6)	304.4 ± 6.6 (7)
[K^+^] (mmol L^−1^)	2.2 ± 0.0 (6)	2.4 ± 0.1 (7)
[Na^+^] (mmol L^−1^)	150.8 ± 1.5 (6)	150.9 ± 1.5 (7)
[Cl^−^] (mmol L^−1^)	127.8 ± 1.8 (6)	133.4 ± 1.7 (7)
[Ca^2+^] (mmol L^−1^)	0.67 ± 0.05 (6)	0.71 ± 0.06 (7)

Plasma osmolality, ion concentrations and blood variables of control (non-perfused) and ½ SW (gastrically perfused with half-strength seawater, ~ 17 ppt) at the last day of perfusion (day 4). Only blood variables obtained from the dorsal aorta are included. Haematocrit, [Haemoglobin] and pH was measured immediately after the end of the protocol. All values are means ± SEM. Sample sizes are indicated within parentheses for each treatment group. There were no significant differences (*P* < 0.05) between treatment groups, as assessed by independent samples *t* tests

## Discussion

### Gastric perfusion with half-strength seawater elevates stroke volume and cardiac output in trout

Here, we show that some of the characteristic circulatory responses that have previously been documented following SW transfer in rainbow trout (Maxime et al. 1991; Brijs et al. 2015) can be elicited by perfusing the gastrointestinal tract of trout maintained in FW with ½ SW fluid. Importantly, the percentage increase in CO between day 0 and 4 was over twofold greater with the ½ SW perfusion relative to the control treatment (+ 35.8% vs. + 74.5%). These increases were exclusively mediated by elevations in SV, which again, were more than twice as large in the ½ SW treatment relative to the control (+ 43.3% vs. + 90.6%). Such SV-mediated elevations in CO are within the range of those previously observed in rainbow trout acutely exposed to full-strength SW (Maxime et al. 1991), as well as after chronic SW acclimation (Sundell et al. 2018; Morgenroth et al. 2019; Brijs et al. 2016a,b). Although the elevated SV observed in SW may be explained by neuro-humoral mechanisms that stimulate cardiac contractility, Brijs et al. (2016b) demonstrated that SW acclimation results in significant increases in central venous pressure and thus cardiac preload. Therefore, it can be speculated that, in this study as well as in SW-acclimated fish, hyperosmolality in the gut induces vascular changes that ultimately result in elevated central venous pressure affecting SV.

### Gastric perfusion with half-strength seawater results in larger changes in gastrointestinal blood flow and vascular resistances in trout

The increase in CO observed in SW-acclimated trout is thought to supply the gastrointestinal tract with additional convectional capacity to extract and transport ions and water around the body as drinking starts (Brijs et al. 2016b; Morgenroth et al. 2019). Although gastrointestinal perfusion with ½ SW elicited pronounced changes in cardiac function, the overall vascular responses were less pronounced. This may indicate that additional stimuli, independent of gastrointestinal luminal salinity, are required to fully elicit the vascular resistance changes typically associated with SW transfer in trout. Nevertheless, these stimuli likely act in concert with internal milieu-sensing mechanisms within the gut, given that some weaker vascular effects were elicited by saline perfusion. For example, although GBF increased across experimental days in both treatment groups, the % Δ from baseline was more than twofold greater in the ½ SW treatment than the control. This resembles the increases in GBF observed in trout acutely and chronically transferred to SW (Brijs et al. 2015, 2016a). This was associated with a trend for a reduced *R*_Sys_ in the ½ SW treatment compared with the control, as well as significantly larger reductions (% Δ from baseline) in *R*_Sys_ and *R*_GI_ in the ½ SW treatment. Again, such reductions in *R*_Sys_ have previously been documented in acutely transferred (Maxime et al. 1991) and SW-acclimated trout (Morgenroth et al. 2019; Sundell et al. 2021; Olson and Hoagland 2008) and, although it has not been measured directly, accumulating evidence indicates that the systemic vasodilation in SW-acclimated trout can, to a large extent, be explained by reduced *R*_GI_ (Morgenroth et al. 2019; Sundell et al. 2018). We interpret these findings as evidence for the existence of mechanisms responsive to osmotic changes within the gut that trigger vasoactive responses, as gastric perfusion with ½ SW elicits several vascular changes that are characteristic of whole-animal SW-acclimation. While we can only speculate regarding the efferent neuro-humoral mechanisms eliciting the cardiovascular changes in response to gastric perfusion, changes in α-adrenergic tone may be involved, since a recent study showed that reduced α-adrenergic vascular tone partially contributes to the lowering of *R*_Sys_ in SW-acclimated trout (Sundell et al. 2018).

On the other hand, if the gastrointestinal perfusate administered in the current study was fully or partially absorbed without a full compensatory increase in urination, a total blood volume increase would have ensued. For the perfused ½ SW fluid to be absorbed it must be coupled to ion uptake, given the osmotic gradient between the perfused fluid and the extracellular fluid/blood. However, plasma osmolality and ion composition were not affected by stomach perfusion, and was within the normal range for rainbow trout (Nordlie 2009). Furthermore, an exacerbated intestinal fluid uptake would likely be reflected in a substantial haemodilution resulting in reduced haematocrit, but this was clearly not the case. This leads to the conclusion that the perfused fluid, if absorbed, was well compensated for via increased urination and ion secretion. Likewise, the lack of change in GBF/CO in the ½ SW perfused fish, which contrasts with the increased GBF/CO observed with SW acclimation (Brijs et al. 2016a), may be explained by fish in FW having to allocate a larger proportion of the total blood flow to the kidney. Indeed, FW teleosts have an elevated glomerular filtration rate to produce large amounts of dilute urine, as opposed to the low volumes of isosmotic urine produced in SW fish (Edwards and Marshall 2013; Marshall and Grosell 2005; Takvam et al. 2021). Therefore, the kidney may receive a larger proportion of blood flow in FW than in SW, especially if the fluid perfused into the stomach is absorbed and glomerular filtration rate has to be elevated further to maintain homeostasis, potentially masking any differences in GBF/CO.

## Conclusions and perspectives

The present study on rainbow trout shows that some key circulatory responses displayed during SW transfer in rainbow trout can be elicited by perfusing the gut directly with ½ SW while maintaining the fish in FW. This points to the existence of internal gastrointestinal sensory mechanisms that respond to changes in luminal osmolality and/or ion concentration, ultimately eliciting cardiovascular responses. Yet, the role of other stimuli than the one tested here cannot be ruled out. For instance, in mammals, gastrointestinal vasodilation can also be induced by intestinal mechanical stimulation (e.g., mucosal stroking and/or intestinal wall distension; Eklund et al. 1980; Biber et al. 1971; Reed and Vanner 2003; Fahrenkrug et al. 1978; Chou and Grassmick 1978), but it is not known whether a similar mechanism exists in fish. Thus, although our data seem to indicate a role of gut hyperosmolality in eliciting cardiovascular responses, we must consider a potential effect of gastrointestinal wall distention generated by the ingested water. Previous studies on two teleost species concluded that neither brief gastric distension (30 min in rainbow trout), nor more prolonged distensions (starting at a volume equivalent to 8–10% body weight and subsequently decreasing the volume over a 72-h period in shorthorn sculpin, *Myoxocephalus scorpius*), had any appreciable effect on GBF, although it did induce substantial elevations in *P*_DA_ and *R*_Sys_ (Seth et al. 2008; Seth and Axelsson 2009). Although these responses are clearly different from the reduced *R*_Sys_ and maintained *P*_DA_ observed here with intestinal perfusion with ½ SW, it is still possible that distension of more distal sections of the gastrointestinal tract could induce different cardiovascular effects. While a single bolus injection (1.2 ml kg^−1^) with 0.9% saline into the proximal intestine of a ~ 500 g rainbow trout had no cardiovascular effects (Seth et al. 2009), it is possible that a larger and more sustained distension, as would possibly occur with continuous drinking in SW, can elicit cardiovascular changes. For example, shorthorn sculpin experience a sharp decrease in *R*_GI_ several hours after force feeding with an isosmotic diet, which might coincide with the entry of food into the proximal intestine (Seth and Axelsson 2009). Although a nutrient-induced hyperaemia is probably the most important contributor to that response (Seth et al. 2009, 2011), it is also possible that distention-induced mechanoreception may play a role. This could be of particular importance in fishes that drink continuously in SW like rainbow trout, because acclimation to SW is known to induce an almost doubling of the diameter of the proximal/middle intestine in rainbow trout (Brijs et al. 2017), and thus warrants further investigation.

Regardless of the stimuli eliciting the cardiovascular effects, our data suggest that internal gastrointestinal milieu-sensing mechanisms with vasoactive effector components, similar to those previously described in mammals, regulate and finetune physiological responses allowing euryhaline fishes to osmoregulate in environments with contrasting salinities. The resultant circulatory adjustments ensure an adequate perfusion of gastrointestinal tissues involved in processing the imbibed fluid to facilitate water and ion fluxes, and possibly to sustain increased metabolic demands of the tissues.

## Supplementary Information

Below is the link to the electronic supplementary material.

Supplementary file1 (XLSX 20 KB)
